# Extracellular vesicles: catching the light of intercellular communication in fibrotic liver diseases

**DOI:** 10.7150/thno.77256

**Published:** 2022-10-03

**Authors:** Yijie Li, Jianzhi Wu, Runping Liu, Yinhao Zhang, Xiaojiaoyang Li

**Affiliations:** 1 School of Life Sciences, Beijing University of Chinese Medicine, Beijing, 100029, China.; 2 School of Chinese Materia Medica, Beijing University of Chinese Medicine, Beijing, 100029, China.

**Keywords:** Extracellular vesicles, fibrotic liver disease, intercellular communication, hepatocyte, hepatic stellate cell

## Abstract

The increasing prevalence of fibrotic liver diseases resulting from different etiologies has become a major global problem for public health. Fibrotic liver diseases represent a redundant accumulation of extracellular matrix, dysregulation of immune homeostasis and angiogenesis, which eventually contribute to the progression of cirrhosis and liver malignancies. The concerted actions among liver cells including hepatocytes, hepatic stellate cells, kupffer cells, liver sinusoidal endothelial cells and other immune cells are essential for the outcome of liver fibrosis. Recently, a growing body of literature has highlighted that extracellular vesicles (EVs) are critical mediators of intercellular communication among different liver cells either in local or distant microenvironments, coordinating a variety of systemic pathological and physiological processes. Despite the increasing interests in this field, there are still relatively few studies to classify the contents and functions of EVs in intercellular transmission during hepatic fibrogenesis. This review aims to summarize the latest findings with regards to the cargo loading, release, and uptake of EVs in different liver cells and clarify the significant roles of EVs played in fibrotic liver diseases.

## Introduction

Liver fibrosis is a common pathogenic process of chronic liver diseases including but not limited to primary biliary cholangitis, hepatitis B virus, alcohol or nonalcoholic steatohepatitis and cirrhosis caused by multiple risk factors like excessive bile acids, alcohol consumption, overloaded lipids and virus infection, and is characterized by excessive accumulation of extracellular matrix (ECM) and the formation of fibrous scar 1, 2. Regardless of the causes of hepatic fibrosis, patients will meet the stage of elevated serum liver transaminase values like AST and ALT, excessive buildup of bilirubin and abnormal imaging of liver parenchyma. In the clinic, liver biopsy is usually performed as a common invasive procedure to accurately assess the severity of different liver diseases but with a lot of complications including but not limited to pain, hemorrhage and bile peritonitis 3. On the other hand, although several non-invasive strategies, such as MRI fibroscans, have been applied for the diagnosis of liver fibrosis, they may be more suitable and accurate for diagnosing advanced fibrotic disorders 4. What is worse, problems with poor targeting and severe side effects limit the clinical application of existing drugs in treating liver fibrosis. Therefore, it is of great significance to deeply excavate the pathogenesis of liver fibrosis and discover novel drug targets for fibrotic liver diseases.

Recently, researchers have paid more attention to the pathological communication between different types of cells inside the liver, which can interact with each other and synergistically trigger a distinct cascade of fibrotic responses. Hepatocytes are usually damaged first and spark inflammation by releasing a variety of signaling substances including damage-associated molecular patterns (DAMPs), which further activate resident liver immune cells and recruit bone marrow-derived immune cells into the injured sites 5. Meanwhile, injured cholangiocytes adjacent to hepatocytes can also release inflammatory cytokines and chemokines and contribute to a pathological reparative reaction 6. Under normal conditions, liver sinusoidal endothelial cells (LSECs) constitute fenestrated capillaries that offer a channel for transporting resident macrophages and natural killer (NK) cells; once damaged or influenced by injured adjacent cells, LSECs start dedifferentiation towards a capillarized phenotype, accompanied with the release of soluble factors that delivery to neighboring cells, such as hepatic stellate cells (HSCs). The involved signaling pathways including but not limited to phosphatidylinositol 3-kinase (PI3K)/protein kinase B (AKT), Wnt and signal transducer and activator of transcription 3 (STAT3) signaling contribute to HSCs transdifferentiate from a quiescent state to an activated state (*i.e.*, myofibroblasts), becoming the core factor driving fibrosis 7-9. Under the stimulation of the above factors, HSCs not only produce ECM but also aggravate liver inflammation by secreting cytokines, emphasizing emerging molecular and cellular signals that drive fibrotic liver injury. Finally, large amounts of ECM components continuously occupy the empty space left by dead cells to induce excessive repair in the liver, which may be initially beneficial, but in turn, prevent damaged liver cells from returning to normal function and then construct a vicious circle 10. Collectively, intercellular interactions among liver cells are multidirectional and work in concert to affect the progression of liver fibrosis. Emerging studies have highlighted the potential of extracellular vesicles (EVs) in the diagnosis and therapy of liver fibrosis *via* modulating intercellular communication networks in the liver.

### EVs: important mediators for intercellular transmission

**Figure 1:**
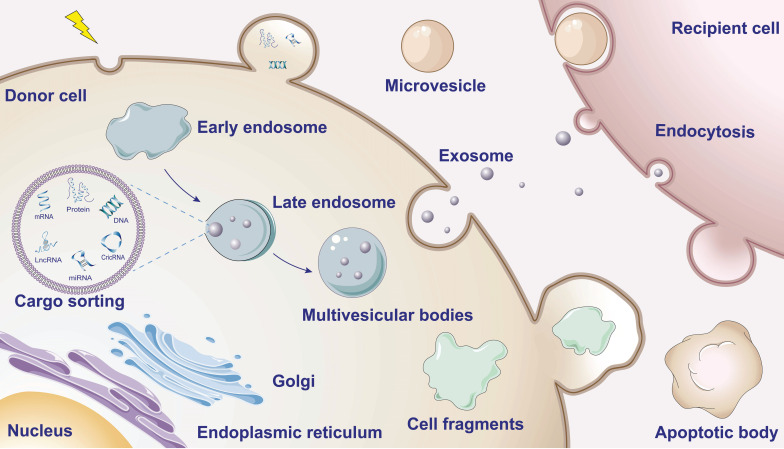
**Schematic overview of EV biogenesis and cargo.** When the donor cells are stimulated, the plasma membranes invaginate to form early endosomes and gradually form late endosomes. The late endosome membranes then invaginate while encapsulating informative material (containing proteins, nucleic acids, lipids, etc.) to mature into multivesicular bodies (MVBs). Eventually, the MVBs fuse with the cell membrane releasing the inner vesicles into the extracellular environment to form exosomes. Outward budding and fission of the plasma membrane and subsequent release of vesicles into the extracellular space form microvesicles. During apoptosis the cytosolic membrane crumples and invaginates, dividing and enveloping the cytoplasm and containing DNA material and organelles to form apoptotic vesicles.

Typically, intercellular communication is roughly classified into following three categories: (i) direct cell contact that mediated by sets of adhesion molecules, (ii) gap junction by establishing tunneling nanotubules between neighboring cells and (iii) transmission of extracellular chemical signal depending on mechanism of ligand-receptor binding in the endocrine manner 11, 12. As membrane-bound vesicles with the structure of typical lipid bilayer, EVs encapsulate signaling molecules and thus afford a more efficient modality of transmitting intercellular signaling during cell-to-cell crosstalk (**Figure 1**). After originated from either healthy or stressed cells, EVs deliver signaling substances including DNAs, RNAs, microRNAs (miRNAs), long non-coding RNAs (lncRNAs), lipids and proteins into recipient cells 13. Interestingly, nucleic acids or proteins specific derived from damaged organelles such as mitochondrial DNA (mtDNA) can also be encapsulated by EVs 14. EVs containing virus-coding RNA and proteins are also reported to secret from the infected cells and transfer to the target cells 15. Based on their different biogenesis, EVs are further classified into three main categories: exosomes, microvesicles and apoptotic bodies, ranging from 30 nm to 1,000 nm in diameter 16. Inward budding of endosomal membrane forms intraluminal vesicles that are the precursor of exosomes and are encased by multivesicular endosomes. After maturation, exosomes are released through the fusion with the plasma membrane of multivesicular bodies 17. Unlike exosomes, microvesicles are only formed by the outward budding at the plasma membrane 18. Characteristically, large-scale blebbing of plasma membrane makes cells dissociate into apoptotic bodies when cells undergo apoptosis, autophagy and programmed necrosis 19. With the gradual deepening research of the cellular communication mediated by EVs, increasing evidence show that EVs play important roles in the occurrence and development of various chronic diseases.

To date, although a few studies have generally summarized the functions of EVs played in different liver diseases, a systematic understanding of potential effects and underlying mechanism of delivering substances among multiple liver cells is still lacking and the complexity and bidirectionality of EVs existing in livers, which may be associated with the direction of material transfer between cells and the stage of fibrosis, has been neglected. Thus, a review from perspectives of donor and recipient cells of EVs will help future researchers to conduct more in-depth studies of EVs-mediated communications. Herein, we comprehensively summarized and discussed recent findings focusing on the physiological and pathological functions of EVs and substance transmitting information* via* EVs among different liver cells. We also probed into the role of EVs in the initiation and progression of fibrotic liver diseases and emphasized the therapeutic potential of targeting EVs in the clinic.

## Hepatocytes-centered EVs network

**Figure 2:**
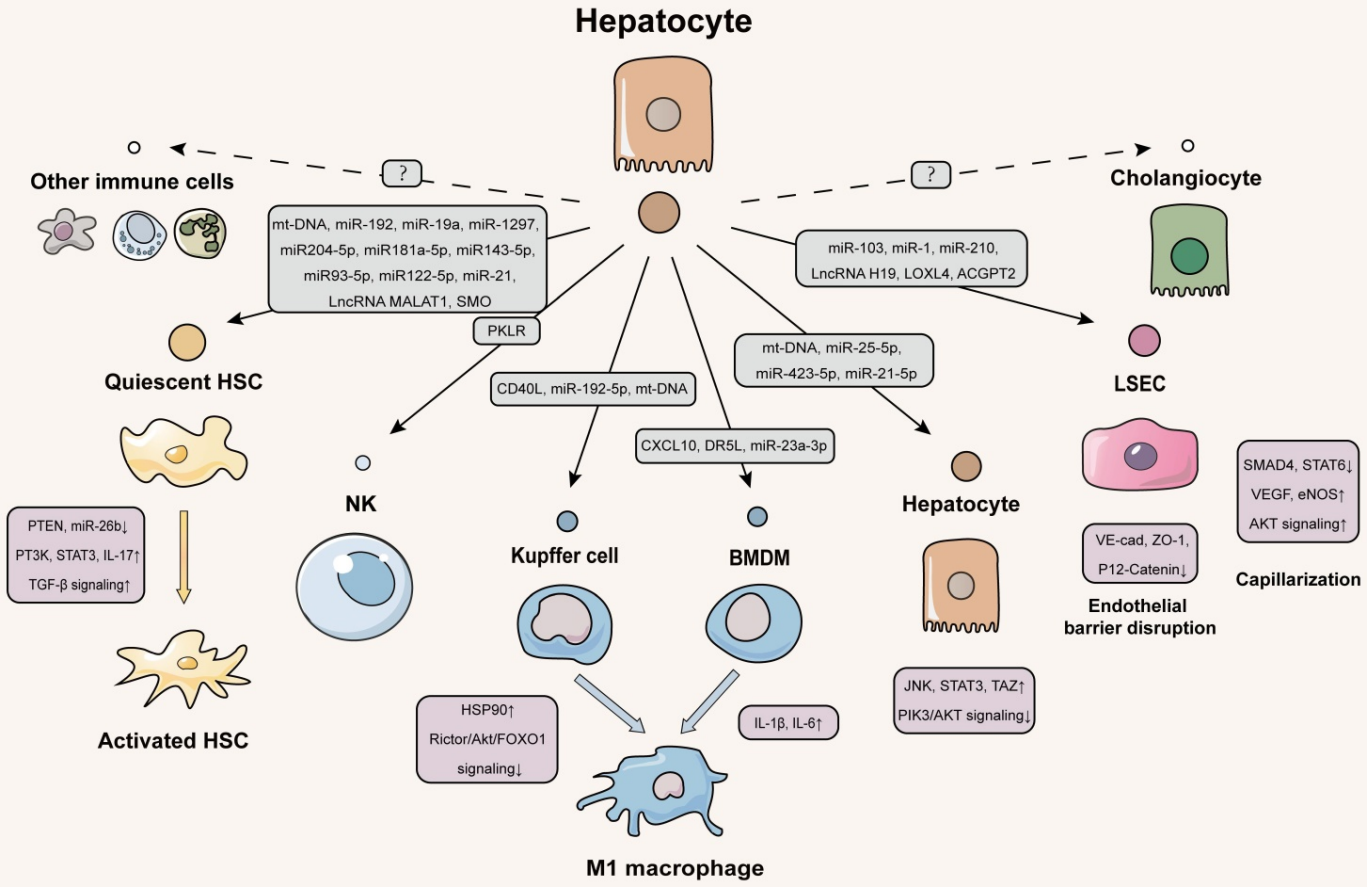
** Schematic diagram of hepatocytes-centered EVs network during liver fibrotic diseases.** The EVs secreted by hepatocytes contain a variety of contents that are delivered to the quiescent HSCs to activate them. Activated HSCs then secrete cytokines that stimulate immune cells to exacerbate liver fibrosis. Both liver resident macrophages (kupffer cells) and BMDM receive EVs from hepatocytes and transdifferentiate into M1-type pro-inflammatory macrophages. Hepatocytes deliver EVs to LSECs to disrupt the integrity of the hepatic blood sinusoidal endothelium and accelerate capillarization to worsen liver fibrosis. Hepatocytes also autocrine EVs to deliver injury signals. Further studies are needed to investigate how cholangiocytes and immune cells other than NK cells communicate with hepatocytes *via* EVs. The solid line indicates that this has now been reported and the dotted line indicates that it has not been reported.

Given that hepatocyte injury is a ubiquitous event of liver fibrosis, question about how hepatocytes exert the regulation of neighboring liver cells *via* EVs will be gradually unveiled by many lines of evidence (**Figure 2**).

### Delivering EVs from hepatocytes to HSCs

The repertoire of HSC functions is recently found to be much more diverse, since they can act as antigen presenting cells (APC), express pattern recognition receptors (PRRs) and respond to hepatocyte-derived DAMPs 20. Furthermore, as is known, a wide variety of stimulus including drugs, virus and lipids can trigger the transmission of EVs between hepatocytes and HSCs, suggesting that they may communicate with each other through the delivery of information, thus affecting the progression of liver fibrosis 21. The transforming growth factor beta (TGF-β) signaling pathway, AKT signaling pathway and hedgehog signaling pathway are dominant pro-fibrotic signaling pathways by regulating the transcription of fibrosis-related genes in livers 22. According to our latest study, mtDNA-enriched EVs released from oxidative stressed hepatocytes induced by either carbon tetrachloride (CCl_4_) or acetaminophen directly activated HSCs and drived liver scarring by upregulating the transcription of fibrotic genes including *Acta2*, *Col1a1* and *Fibronectin* 5. Although we did not explicitly investigate how these mtDNA-enriched EVs regulated gene transcription because of the mtDNA loop structure, there are many other studies linking this change to miRNAs, another kind of non-coding RNAs being recognized to regulate a variety of target genes. Recent study showed that after infected with hepatitis C virus (HCV), miR-192 was released from hepatocytes *via* exosomes and further transmitted into HSCs, resulting in the trans-differentiation of HSCs into myofibroblasts, which was mediated by the production of TGF-β1 and subsequently upregulation of fibrogenic markers such as *Acta2* and *Col1a1* 23. Similarly, Devhare PB *et al.* reported that exosomes-containing miR-19a released from hepatocytes in the same injured situation were also delivered into HSCs and depleted suppressor of cytokine signaling 3 (SOCS3) to activate signal transducer and activator of transcription 3 (STAT3)-mediated TGF-β signaling pathway, which in turn promoted the HSC activation 24. Moreover, HCV-derived EVs were reported to contain lower levels of antifibrogenic miRNAs including miR204-5p, miR181a-5p, miR143-5p, miR93-5p and miR122-5p, and higher levels of miRNAs associated with HCV-related immunopathogenesis including miR-222-3p, miR-146a, miR-150-5p, miR-30c, miR-378a-3p and miR-20a-5p, which induced fibrogenic marker expression (*Fn-1*, *Acta2*, *Col1a1* and *Tgfb1*) and HSC activation 25, 26. Lipids generally deposit in hepatocytes as an adaptive response and further increase free fatty acids (FFAs), while the dysregulation of lipid homeostasis transmits damage signals to other liver cells *via* the release of EVs. Lee YS *et al.* further confirmed the significant functions of miR-192 during lipids-induced fibrosis. Notably, palmitic acid increased the number of isolated exosomes from hepatocytes and then directly transfected exosomal miR-192 into HSCs to enhance the expression of fibrosis-associated genes including *TGFb1*, *Acta2* and *Col1a1*, which accelerated the progression from steatosis to non-alcoholic steatohepatitis (NASH) 27. As another pro-fibrotic pathway, AKT signaling can be negatively regulated by the gene of phosphate and tension homology deleted on chromosome ten (PTEN) that contributes to the proliferation of HSCs 28. Exosomal miR-1297 secreted from lipotoxic hepatocytes was reported to decrease the mRNA and protein levels of PTEN and increased the phosphorylation of PI3K and AKT in HSCs, which then triggered HSC activation and proliferation 29. In addition, exosomal miR-21 released by hepatoma cells converted normal HSCs to cancer-associated fibroblasts (CAF) by directly downregulating PTEN and then activating 3-phosphoinositide-dependent kinase 1 (PDK1)/AKT signaling pathway. Once activated, CAF further promoted angiogenesis and fibrotic injury by secreting angiogenic cytokines, such as vascular endothelial growth factor (VEGF), matrix metalloproteinase 2 (MMP2), MMP9 and TGF-β 30. It is worth noting that miRNAs not only can be transported *via* EVs, but also be regulated in the recipient cells by other substances delivered from EVs. Exosomes containing lncRNA metastasis-associated lung adenocarcinoma transcript 1 (MALAT1) were derived from hepatocytes treated with arsenite and were further transported into HSCs to promote their activation through the downregulation of miR-26b and subsequent upregulation of *Col1a2* 31. In addition, activation of HSCs has a strong correlation with hepatocellular carcinoma (HCC) occurrence and development. As an oncogene in HCC, smoothened (SMO) was packaged in HCC-derived exosomes, transmitted to HSCs and triggered HSC activation by increasing GLI family zinc finger 1 expression and activating hedgehog pathway. Furthermore, SMO led to the transcriptional activation of miRNA let-7b host gene, which in turns, upregulated the expression of SMO and formed a profibrotic and vicious feedback loop 32.

An additional point deserved to be mentioned is that hepatic pathological events caused by exosomes delivering from hepatocytes to HSCs may be not only limited to these two types of cells. A previous study confirmed that exosomes derived from CCl_4_-treated hepatocytes specifically activated toll-like receptor 3 (TLR3) and upregulated interleukin-17A (IL-17A) expression in HSCs, leading to the production of IL-17, IL-1β and IL-23 at the early stage of fibrotic liver injury. In addition, these increased cytokines in exosome-treated HSCs could subsequently promote the recruitment of γδT cells and boosted IL-17A production in γδT cells by increasing the expression of retinoic acid receptor-related orphan receptor γt (RORγt) 33. Considering that the apoptosis and exhaustion of HSCs are closely associated with the activation of CD4^+^ T helper cells, CD8^+^ cytotoxic T cells and other innate T cells, we speculate more extensive changes happened in the 'bystander' immune cells during the communication between hepatocytes and HSCs.

### Delivering EVs from hepatocytes to immune cells

Under pathological conditions, hepatocytes produce different chemokines at the site of injury, recruiting and triggering more immune cells to induce local damage reactions 34. As a vital category of immune cells, macrophages ubiquitously exist and are susceptible to be affected by hepatocyte-derived EVs due to their chemotactic activities 35. Typically, macrophages can be roughly divided into liver-resident macrophages (kupffer cells) or recruited macrophages based on their origin. Besides, two dynamic active states of macrophages are usually portrayed as classical activated M1 and alternative activated M2 phenotypes, the former of which promote inflammation and the latter ones accelerate fibrosis progression 36. Numerous studies have proved that, regardless of the source or phenotypes, macrophages have to take risks of EVs released by injured hepatocytes. It has been reported that kupffer cells were evoked by EVs released from FFAs- and cobalt chloride-stimulated hepatocytes and further secreted pro-inflammatory cytokines to promote liver fibrosis 37. Saha *et al.* intensively studied those data and found that EVs secreted from primary hepatocytes of mice with fibrotic liver disease could increase the percentages of kupffer cells (inflammatory M1 type) and infiltrating monocytes by upregulating the expression of heat shock protein 90 (HSP90) 38. In addition to secreting pro-inflammatory cytokines, EVs released from lipotoxic hepatocytes also play a vital role in the activation and transformation of kupffer cells. Under the stimulation of large amounts of alcohol, exosomal CD40 ligands were released from hepatocytes as results of caspase 3/7 activation and were further delivered into kupffer cells, in which promoted the polarization of M1-type inflammatory phenotype and triggered continuous inflammation 39. A subsequent study further explored the possible mechanisms and demonstrated that exosomal miR-192-5p released from palmitate-induced lipotoxic hepatocytes promoted the pro-inflammatory M1 phenotype activation, the differentiation of THP-1 macrophages and liver fibrosis at least partially by downregulating the Rictor/AKT/Forkhead box transcription factor O1 signaling pathway 40.

Comparatively, exosomes from impaired hepatocytes appeared to affect peripheral monocytes in a more complex manner than those from *in situ* liver macrophages. It was reported that the activated endoplasmic reticulum to nucleus signaling 1 (ERN1) promoted the transcription of serine palmitoyl transferase genes in mouse hepatocytes *via* upregulating X-box binding protein 1 (XBP1), resulting in ceramide biosynthesis and release of ceramide-enriched inflammatory EVs, which recruited bone marrow-derived macrophages (BMDMs) to the liver, resulting in inflammation and fibrotic injury in mice with diet-induced steatohepatitis 41. Palmitate treatment also promoted the release of EVs containing C-X-C motif ligand 10 (CXCL10) from hepatocytes by activating mixed lineage kinase 3 (MLK3) signaling cascade. Subsequently, the lipotoxic hepatocyte-derived EVs were delivered into BMDMs, and promoted macrophage chemotaxis into the liver in a CXCL10-dependant manner 42. In addition to affecting the chemotaxis of BMDMs, EVs secreted from hepatocytes also have impact on the BMDM activation. Either palmitate or its active metabolite lysophophatidylcholine could promote the release of death receptor 5 (DR5) ligand-bearing EV from injured hepatocytes by activating DR5-caspase 8-caspase 3-Rho-associated kinase 1 (ROCK1) proapoptotic signaling. These lipotoxic EVs further induced BMDM (M1-like phenotype) activation by increasing the gene expression and release of pro-fibrotic cytokines in a receptor-interacting protein kinase 1-dependent manner, accelerating the progression of fibrotic liver injury and inflammation 43. These findings clearly link injured hepatocytes-released EVs to a profibrotic macrophage response.

In addition to the direct dialogue with macrophages, hepatocytes also affect many other immune cells like multifarious T-cells that communicate with macrophages and influence the fibrotic response in the liver. A recent study revealed that under alcoholic stress, hepatocytes were damaged first and secreted mitochondrial double-stranded RNA-enriched exosomes, which were uptaken by kupffer cells and stimulated IL-1β production through the activation of TLR3. The IL-1β produced by those injured kupffer cells further promoted the IL-17A production in γδ T cells in a short time and increased IL-17A production in CD4^+^ T cells and liver fibrosis after chronic alcohol consumption 44. In addition to CD4^+^ T cells, CD8^+^ T cells are another crucial type of T lymphocyte involved in intercellular communication-mediated immune responses. Moreover, hepatoma cells-derived exosomal miR-23a-3p were uptaken by THP-1 monocytes-derived macrophages, which subsequently decreased PTEN expression and elevated AKT phosphorylation and PD-L1 expression in macrophages. The macrophages further decreased the ratio of CD8^+^ T cells, promoted T cell apoptosis and fibrotic liver damage 45. Recently, Liu *et al.* also identified the interaction between hepatocytes and NK cells and demonstrated that hepatic depletion of FBP1 derepressed an zeste homolog 2 (EZH2)-dependent transcriptional program to inhibit PKLR expression. This led to the reduced transmission of PKLR-enriched EVs from hepatocytes to NK cells, thus dampened the function of EV-targeted NK cells and promoted liver fibrosis and tumorigenesis 46. Collectively, at present, the research on the intercellular transmission of hepatocyte-derived EVs mainly focuses on their interaction with immune cells represented by macrophages and associated T cells, prompting the specific EVs to be activated and produce diverse cytokines to disturb immune microenvironment and thus accelerate hepatic fibrogenesis.

### Delivering EVs from hepatocytes to liver sinusoidal endothelial cells (LSECs)

Following LSECs injury, the pathological generation of intrahepatic vessels and progressive microvascular dysfunction will increase hepatic vascular resistance, leading to the development of portal hypertension that regarded as a major complication of liver fibrosis 47. Previous study reported that hedgehog ligands-enriched EVs released from myofibroblastic HSCs altered the phenotype of neighboring LSECs and promoted vascular remodeling by upregulating the expressions of SEC activation markers including B-Actin, annexin V and keratin 19 (CK19) and inducing the release of vasoactive factors like nitric oxide (NO) 48. Numerous reports of fibrotic liver diseases pointed out that significant changes in miRNA profiles played a destructive effect on hepatic sinusoid since individual miRNA simultaneously regulated multiple target genes. As a molecular cargo of EVs secreted by hepatoma cells, miR-103 was reported to significantly disrupt endothelial barrier integrity by suppressing adherent molecules including VE-Cadherin, p120-catenin and zonula occludens 1 (ZO-1) in LSECs, which increased vascular permeability and promoted fibrotic liver injury 49. Although human umbilical vein endothelial cells (HUVECs) are not obtained from the liver, they are commonly used in *in vitro* experiments as a replacement of LSECs due to their relative ease of isolation and similarity in characteristics to LSEC. MiR-1 was enriched in exosomes secreted by steatotic hepatocytes and caused inflammatory and fibrotic injury in HUVECs through the following two pathways: on the one hand, exosomal miR-1 activated the expression of a series of genes associated with endothelial inflammation and fibrosis, especially for vascular cell adhesion molecule-1; on the other hand, it suppressed the expression of the zinc finger protein Krüppel-like factor 4 (KLF4) in HUVECs 50. It is well-known that small mothers against decapentaplegic (SMAD) proteins function as important intracellular signaling molecules in TGF-β signaling and are only substrates for TGF-β intracellular kinase, which is the reason why SMADs are critically linked to the development of liver fibrosis 51. Notably, KLF4 silencing effectively 'hijacks' SMAD promoters to inhibited TGF-β transcription 52. Considering the close relationship between KLF4 and SMAD, the researchers also examined the role of SMAD between hepatocytes and HUVEC. Exosomal miR-210 was released from hepatoma cells and transferred to HUVECs, thereby promoting the formation of capillary-like structures. Furthermore, exosomal miR-210 increased the microvascular density and promoted liver fibrosis *in vivo*, which was primarily due to the inhibition of SMAD4 and STAT6 expression 53. Our prior studies identified that lncRNA H19 rapidly and highly expressed in the liver of patients with liver fibrosis and was closely associated with the progression of liver fibrosis 54. Interestingly, exosomal lncRNA H19 was reported to promote HUVECs proliferation and adhere to CD90^+^ cancer stem cells (CSCs) by regulating intercellular cell adhesion molecule-1 phenotype and further regulate hepatic material exchange microenvironment. In addition, after entered HUVECs, exogenous H19 exosomes activated the production and secretion of VEGF and thus increased tubular-like structures in the liver sinusoids, leading to the pathological neovascular response and liver fibrosis 55.

In addition to miRNAs and lncRNAs, hepatoma cells can also deliver protein signals *via* EVs to LSECs and accelerate fibrosis progression. Lysyl oxidase like 4 (LOXL4), regarded as extracellular copper-dependent enzymes that involved in ECM cross-linking, was found to be secreted by HCC cells and partly assembled by exosomes to increase the adhesion of cellular matrix *via* phosphorylation of Src and focal adhesion kinase. In addition, HCC-derived exosomes transferred LOXL4 proteins to both HCC cells and HUVECs and promoted angiogenesis and liver fibrosis in a paracrine manner 56. Another research suggested that HCC cell-derived exosomes promoted the angiogenesis of HUVECs by transmitting angiopoietin-2 (ANGPT2) to HUVECs, which in turn, promoted the progression of liver fibrosis. Of note, exosomal ANGPT2 increased the phosphorylation of AKT, endothelial nitric oxide synthases and β-catenin rather than activated the classic ANGPT2/tunica interna endothelial cell kinase (Tie2) pathway in HUVECs 57.

### Delivering EVs from hepatocytes to hepatocytes

Our recent study found that mtDNA-EVs secreted by damaged hepatocytes aggravated hepatocyte death in the CCl_4_-induced hepatic fibrosis mouse model, thus releasing more immunogenic DAMPs and accelerating innate immune response and fibrogenesis in an autocrine manner 5. However, few studies exist evaluating how damaged liver cells affect other hepatocytes to influence the progression of liver fibrosis, which is worthy of further study.

An important proportion of HCC cases are developed from hepatic fibrosis or cirrhosis 58. It is worthy to note that HCC cells also regulate the fibrotic process through the transfer of EVs and feed back to favor their own growth at the same time. Seo W. noticed this phenomenon and first demonstrated that aldehyde dehydrogenase 2 (ALDH2) deficiency exacerbated the development of alcohol-related liver fibrosis in both patients and mouse models developed with liver fibrosis and HCC. Mechanically speaking, a large number of harmful mtDNA were released by ALDH2-deficient hepatocytes through EVs after chronic alcohol exposure, which could be transferred to adjacent HCC cells and subsequently activate multiple carcinogenic pathways such as c-Jun N-terminal kinase (JNK), STAT3, B-cell lymphoma-2 and PDZ-binding motif (TAZ), thus promoting fibrotic liver injury and tumor growth 59. More interestingly, exosomal miRNA-25-5p from HCC cells (HuH-7 or HCCLM3) were able to transfer to anoikis-resistant HCC cells, a kind of liver circulating tumor cells (CTCs) being particularly prone to distant metastases. The mRNA profiles of anoikis-resistant HCC cells revealed the significant reduced leucine-rich repeat containing 7 (LRRC7) expressions since extracellular mature exosomal miR-25-5p directly targeted its 5' UTR, which facilitated the trans-endothelial migration of CTCs in a dose dependent manner 60. HCC cells received self-derived miRNAs (such as miR-423-5p and miR-21-5p)-enriched exosomes to promote cell growth, migration and fibrosis. Moreover, Vps4A utilized exosomes as mediators to downregulate the secretion and uptake of miRNAs in hepatoma cells through the suppression of PI3K/AKT signaling, leading to significant repression of HCC metastasis and fibrotic liver damage 61.

## Delivery of EVs from HSCs to other cells: the core session but has not been well studied

### Autocrine of HSCs-derived EVs

**Figure 3:**
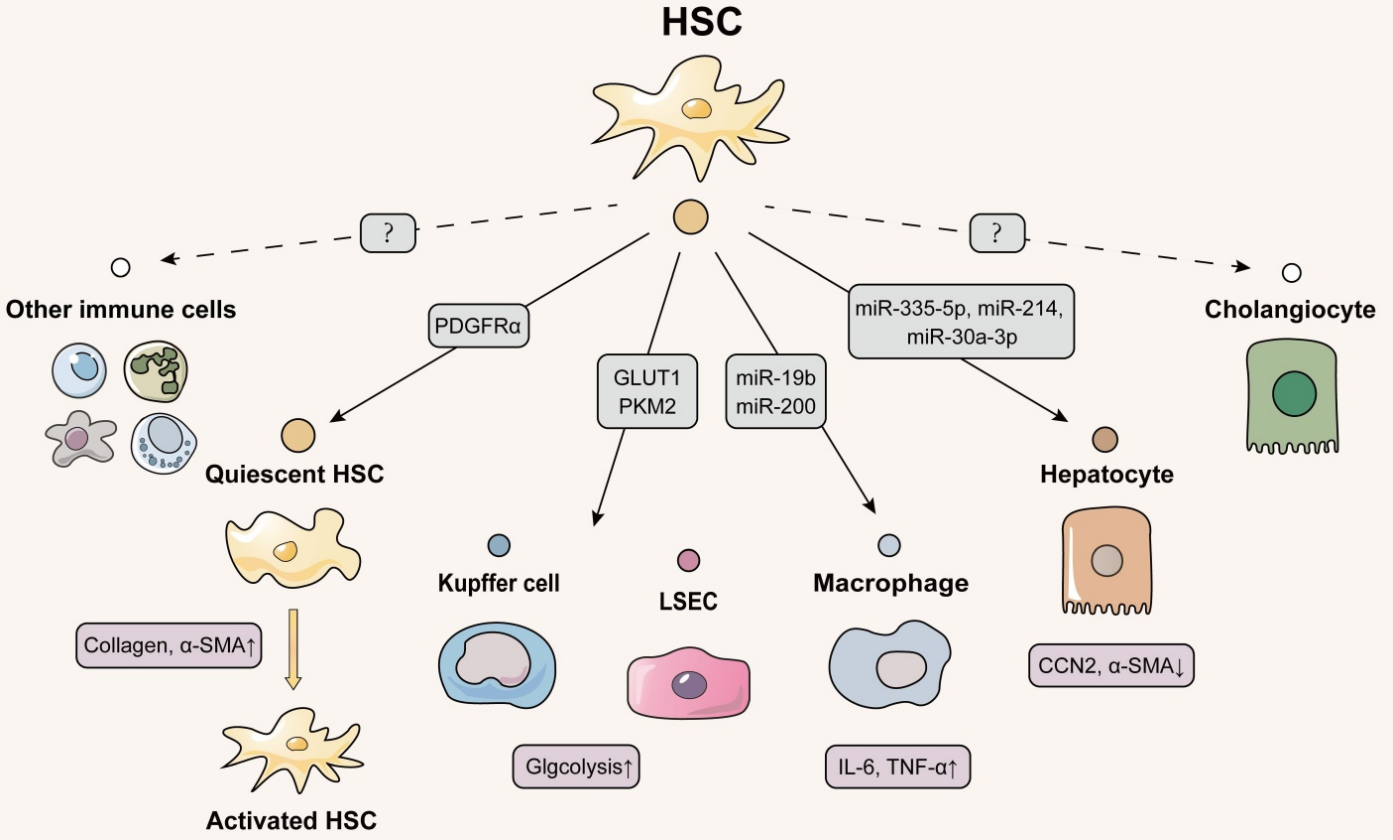
** Schematic diagram of HSCs-centered EVs network.** Activated HSCs deliver EVs to quiescent HSCs to activate the liver fibrosis process. Kupffer cells, LSECs and hepatocytes all receive EVs secreted by HSCs, whereas the effect of EVs released by HSCs on cholangiocytes and other immune cells has not been identified and deserves further investigation, since HSC activation is an important link in the initiation of liver fibrosis. Solid lines indicate that this has now been reported and dashed lines indicate that it has not yet been reported.

Interestingly, despite being the principal fibrogenic cell type in the liver, there are few studies on intercellular communication between HSCs and themselves (**Figure 3**). Notably, compared with paracrine mode, whether HSCs could secrete substances to mediate self-change is an interesting and emerging topic. In addition to the TGF-β we are familiar with, platelet-derived growth factor (PDGF)-BB has also been reported to be an essential factor that activates HSCs. Being activated by PDGF-BB, HSCs secreted phosphorylated tyrosine 720-dependent PDGF receptor α (PDGFRα)-enriched EVs to neighboring HSCs. Moreover, the phosphorylated tyrosine 720-recruited Src homology 2 domain tyrosine phosphatase 2 (SHP2) was also enriched in PDGFRα-loaded EVs and then these EVs were translocated into recipient HSC, triggering the transcription of downstream pro-fibrotic genes (*Col1a1* and *Acta2*) and resulting in the deterioration of liver fibrosis 62. Similarity, Gao* et al*. also demonstrated that PDGF- and SHP2-stimulated HSCs released nano-sized vesicles enriched with fibrogenic proteins including fibroblast growth factor receptor 3 (FGFR3) and Tie2. Then the secreted vesicles further induced HSC migration by upregulating mTOR and Rho-associated protein kinase 1 (ROCK1) signaling and inhibiting autophagy 63. Given that HSC is the key cell type in the hepatic fibrogenesis process, future studies should pay more attention to the autocrine-dependent activation of HSC in different types of liver fibrosis injury, which is of great significance for the targeted inhibition of HSCs.

### Delivering EVs from HSCs to other liver nonparenchymal cells

Exosomes derived from HSCs not only act as mediators of HSCs self-communication but also participate in the dialogue between HSCs and liver nonparenchymal cells. It has been reported that miR19b- and miR200-enriched EVs released from activated-HSCs were transmitted and colocalized to macrophage cell membranes, stimulated the release of pro-fibrotic cytokines (like IL-6 and tumor necrosis factor-α) from these macrophages, prompting hepatic inflammation and fibrosis progression 64. In addition, under stimulation of Hif-1, an oxygen-sensitive transcription factor, activated HSCs released glycolysis-related proteins (GLUT1 and PKM2)* via* exosomes to accelerate glycolysis in liver nonparenchymal cells under hypoxic conditions. Quiescent HSCs highly proliferated due to metabolic phenotype alteration after accepting exosomes 65, while other recipient cells like kupffer cells and LSECs warranted further elucidation.

### Delivering EVs from HSCs to hepatocytes

It has been reported that HSCs phagocytosed apoptotic bodies from hepatocyte, suggesting that the fate of hepatocytes is closely related to HSCs 66. As demonstrated by Coulouarn and his colleagues, after coculturing with activated HSC, HepaRG turned into the migratory and prominent inflammatory phenotype by improving the production of pro-inflammatory cytokines (IL-1β, IL-10 and IL-8) and growth factors (AREG, EREG) and upregulation of acute phase proteins (CP and SAA1) 67. Surprisingly, recent studies have found that EVs secreted by activated HSC can be an effective intervention strategy for suppressing HCC cells growth. HSC-derived EVs loaded with miR-335-5p were uptaken by cancerous hepatocytes and consistently downregulated 13 downstream mRNA targets of miR-335-5p to inhibit hepatocyte proliferation *in vitro*. Besides, exosomal miR-335-5p was indeed utilized by cancer cells to slow the growth of HCC cells after being artificially injected to tumor tissue *in vivo*, suggesting miR-335-5p as a potential HCC therapeutics 68. Moreover, LX-2 cells could rapidly shuttle exosomal MiR-214 to hepatocytes that suppressed connective tissue growth factor (CCN2) 3'-UTR activity and expression of CCN2 and its downstream targets such as α-SMA and collagen, inhibiting fibrogenic signaling 69. More interestingly, a recent study reported that exosomal miRNAs from donor cells are uptaken by recipient cells and further reduces the release of homologous miRNA from recipient cells. Exosomal miR-30a-3p derived from HSCs directly inhibited the migration and invasion of HCCs and indirectly reduced EV-containing miR-30a-3p released from HCC cells by downregulating synaptosome-associated protein 23. Reduction of EV released from HCC cells formed a positive feedback loop to increase miR-30a-3p accumulation in HSCs, which in turn, enhanced the role of miR-30a-3p played in reducing the migration of HCC cells 70.

## The role of LSECs-released EVs in the intercellular communication: main emphasis of future study

### Delivering EVs from LSECs to hepatocytes

Just as hepatocytes generally secrete exosomes to LSECs, hepatocytes can also internalize exosomes derived from LSECs, supporting the relevance between hepatocytes and LSEC during the progression of hepatic fibrosis. A variety of studies have already pointed that LSECs also release exosomes targeting cancerous hepatocytes and thus directly accelerate liver fibrosis. Yes-associated protein (YAP) is a critical downstream target in the Hippo signaling pathway, which may be involved in liver fibrosis by regulating HSC proliferation and apoptosis 71. Several studies have shown that the clinical application of YAP inhibitor verteporfin induced the spread of HCC, of which possible mechanism was further revealed by the study of Yang L and colleagues. Under the deficiency of YAP caused by intervention with small interfering RNAs and verteporfin, vascular endothelial cells exhibited a higher level of vesicle-associated membrane protein 3 (VAMP3) and further promoted the release of exosomes transferring to HCC cells. Notably, these YAP1-deficiency exosomes containing lncRNA MALAT1 increased the invasion and migration of HCC cells through the activation of extracellular signal-regulated kinase 1/2 signaling 72. As mentioned earlier, exosomal MALAT1 released by hepatocytes can directly deliver to the HSCs, so whether MALAT1 released from LSECs *via* EVs also targets and regulates HSCs may be a direction worth exploring in the future.

Meanwhile, unlike 'bad' exosomes secreted by injured or cancerous hepatocytes targeting LSECs during liver fibrosis; 'good' exosomes might be also produced from virus infected LSECs and further deliver to hepatocytes and restore liver function. Giugliano S. first demonstrated that HCV induced the broad transcription of Type I and Type III interferons (IFN) in human LSECs that internalized HCV in a clathrinid-dependent manner. Both Type I and Type III IFNs stimulated human LSECs to release exosomes, which abrogated HCV infection in uninfected human LSECs via robustly upregulating the transcription of Type III IFNs and viperine. Subsequently, LSECs leaked-exosomes dose-dependently upregulated the transcription of IFN-stimulated genes to inhibit of HCV replication in hepatocytes and promoted anti-viral responses, consequently leading to the prevention of liver fibrosis 73.

### Delivering EVs from LSECs to HSCs

**Figure 4:**
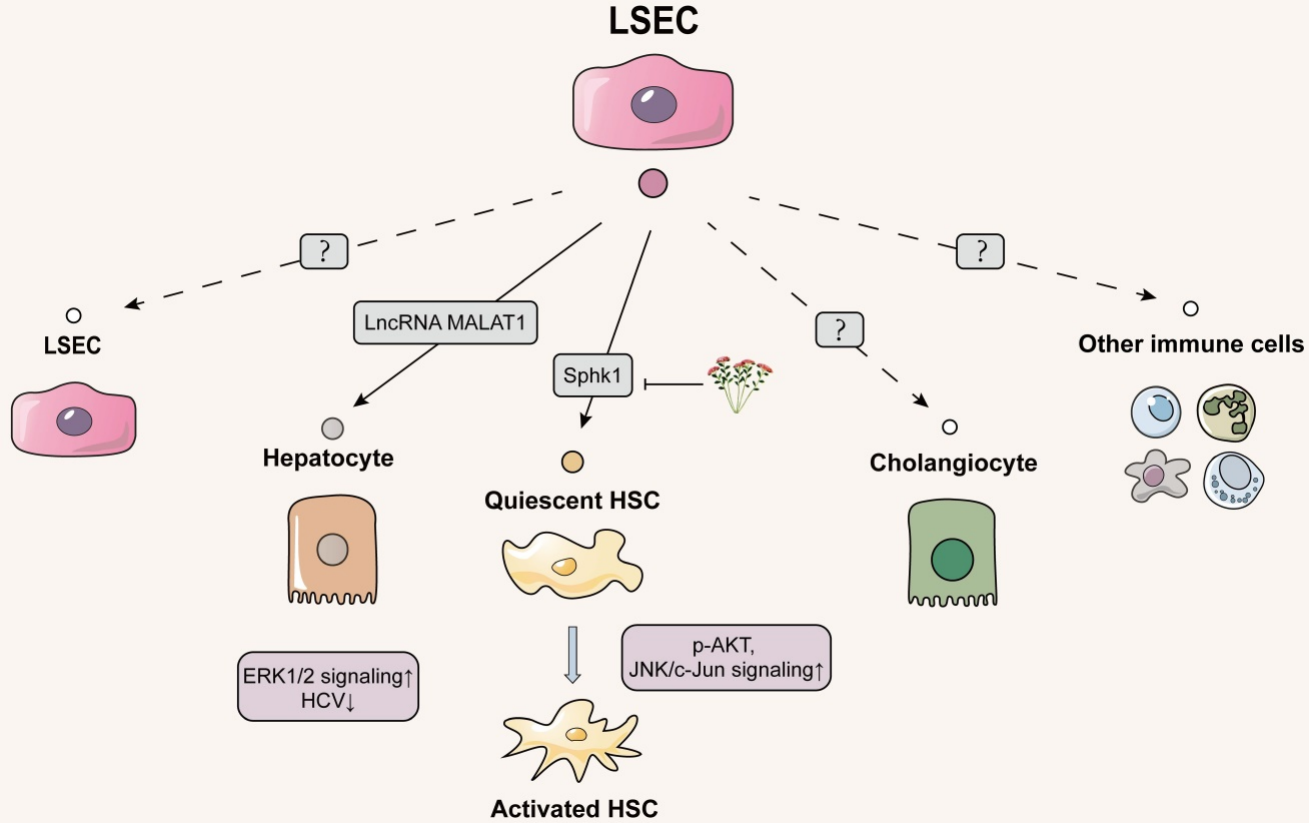
** Schematic diagram of LSECs-centered EVs network.** LSEC-secreted EVs are poorly studied and focus on hepatocytes and HSCs. EVs delivering to hepatocytes both are 'good' or 'bad', with the former inhibiting HCV replication in hepatocytes and the latter activating oncogenic hepatocyte migration. LSECs release pro-fibrotic EVs to HSCs, which can be inhibited by the natural product salidroside. However, the effect of EVs secreted by LSEC on themselves, cholangiocytes and other immune cells has not been explored yet. The solid line indicates that this has now been reported and the dashed line indicates that it has not been reported.

HSCs situate in the subendothelial space of Disse and are anatomically adjacent to LSECs 74. Therefore, the neighboring intercellular communication between liver endothelial cells and HSCs through different transmission modes especially for EVs cannot be ignored. Initially, the possibility of crosstalk between LSECs and HSCs was observed to be associated with sphingosine kinase 1 (SphK1). Endothelial cells-derived exosomes expressed fibronectin on membrane surface and then adhered with α5β1-integrin on HSCs to enter the HSCs through dynamin 2-dependent endocytosis. SphK1 entrapped within endothelial cell-derived exosomes activated AKT phosphorylation while promoted pathological HSC migration and aggravated fibrogenesis 75. Another study further found that natural products influenced the above pathways to alleviate the process of liver fibrosis, thus clarifying the importance of SphK1 in LSEC-to-HSC communication. Salidroside subdued the release of exosomal SphK1 from LSECs and lessened HSC migration and activation by inhibiting AKT phosphorylation. Additionally, it inhibited JNK phosphorylation to reduce mitochondria-mediated hepatocyte apoptosis and apoptotic irritants release, which subsequently decreased the secondary damage to HSCs 76. Most recently, Wu *et al.* first reported that adipocyte fatty acid binding protein (A-FABP) from LSECs exacerbated the bile duct ligation (BDL)-induced liver fibrosis by activating HSCs. Mechanistically, the enhancement of intracellular A-FABP in LSECs activated Hedgehog signaling pathway to potentiate LSEC capillarization. In addition, A-FABP released from the conditional medium (containing an amount of EVs) of LSECs was phagocytized by HSCs in a paracrine manner and then activated the JNK/c-Jun signaling to increase the transactivation of TGFβ1, leading to the perpetuation of HSC activation 77. Although Wu *et al.* didn't distinguish the pro-fibrotic effects of LSEC-released A-FABP on HSCs are dependent on EVs or other ingredients in conditional medium, they still provided a critical future research direction for this field (**Figure 4**).

## Cholangiocytes-centered intercellular communication *via* EVs: less but important

### Delivering EVs from cholangiocytes to hepatocytes

**Figure 5:**
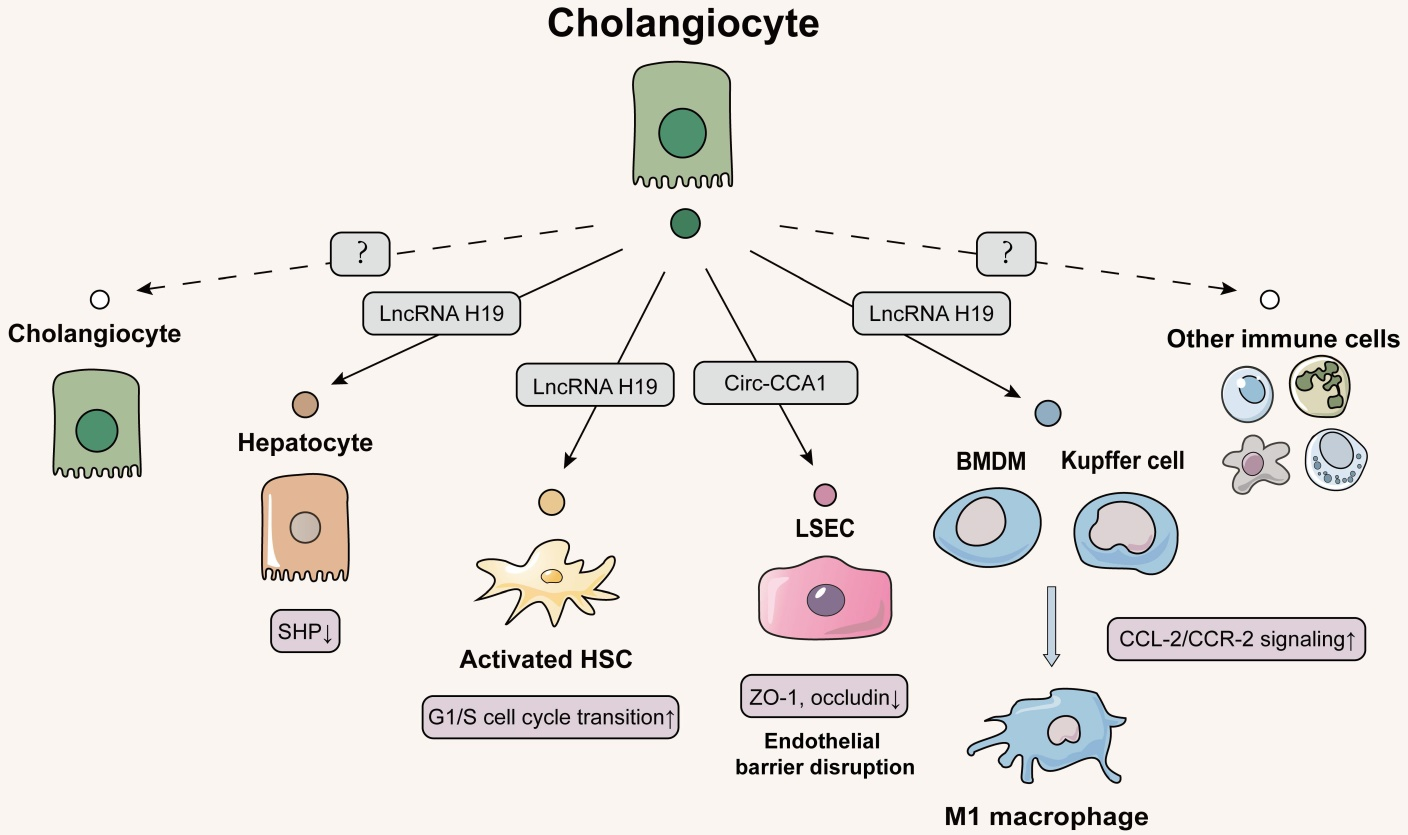
** Schematic diagram of cholangiocytes-centered EVs network.** The cholangiocytes can release lncRNA H19 into hepatocytes, HSCs and macrophages (liver-resident and bone marrow-derived), causing hepatocyte injury, HSC proliferation activation, and macrophage differentiation to the M1 phenotype and exacerbating hepatic fibrosis. Cholangiocyte-derived exosomes are also transmitted to LSECs, which results in the disruption of hepatic blood sinusoidal endothelial barrier by increasing cell permeability. The solid line indicates that this has now been reported and the dashed line indicates that it has not been reported.

Our results and a growing number of other studies all point out that cholangiocytes are the primary targets of exogenous stimulus especially for bile acids and play a very important role in the whole spectrum of liver fibrosis 78. However, there has been little research exploring the roles of EVs isolated from cholangiocytes on liver fibrosis likely due to few quantities and isolation difficulties (**Figure 5**). Under the co-stimulation of estrogen and taurocholate (TCA), lncRNA H19 was markedly induced and enriched in exosomes released from cholangiocytes, which were further uptaken by hepatocytes. Furthermore, we found that overexpressed lncRNA H19 suppressed the level of small heterodimer partner (SHP) in hepatocytes by inhibiting its promoter activity and mRNA stability, thereby disrupting bile acid metabolism and resulting in liver fibrosis 54.

### Delivering EVs from cholangiocytes to HSCs

On the account of strong correlation between hepatocytes and HSCs, we further probed whether lncRNA H19-riched exosomes secreted from cholangiocytes directly regulated the activation or proliferation of HSC and thus affected collagen deposition. As expected, our results showed that cholangiocytes-derived exosomal lncRNA H19 was rapidly uptaken by HSCs and HSC-derived fibroblasts under TCA challenge, which subsequently upregulated the expressions of fibrotic genes to activate HSCs and promoted the trans-differentiation from HSCs to fibroblasts. In addition, exosomal lncRNA H19 also directly promoted HSC proliferation by enhancing G1/S cell cycle transition, leading to the exacerbation of liver fibrosis 79. Meanwhile, it has been shown that lncRNA H19 was increased in serum EVs from patients with biliary atresia and fibrosis and restrained the bioavailability of miRNA let-7 family to promote bile ducts proliferation and BDL-induced liver fibrosis 80. However, further studies are needed to confirm whether lncRNA H19-riched exosomes affect the transcription of fibrosis genes in HSCs by influencing other transcription factors or miRNAs such as let-7.

### Delivering EVs from cholangiocytes to LSECs

During processes of various cholangiopathies, injured cholangiocytes increase the release of various inflammatory and fibrogenic mediators and engage in adjusting barrier function of endothelial cells. An elegant study has shown the potential of intercellular communication between cholangiocytes and LSECs *via* EVs. Cholangiocarcinoma-associated circular RNA 1 were wrapped in EVs secreted by cholangiocytes and transferred to vascular endothelial cells to increase the expression of SH3 domain-containing GRB2-like protein 2 and trimethylation of promoter histone 3 lysine 27, which inhibited the expressions of ZO-1 and occludin and increased the permeability of endothelial monolayer cells, leading to the destruction of vascular endothelial barrier integrity, angiogenesis and inflammatory reaction during liver fibrosis 81.

### Delivering EVs from cholangiocytes to immune cells

In addition to LSEC, immune cells including kupffer cells are also recruited to the injured biliary ductal milieu, promoting cholestasis and fibrotic liver injury. We continued to delve into this question about how lncRNA H19 influenced the pathological microenvironment and liver fibrosis *via* EVs. Notably, we first demonstrated that lncRNA H19-enriched exosomes enhanced the active M1 polarization of kupffer cells and promoted the recruitment and differentiation of BMDMs through the activation of CCL-2/CCR-2 signaling pathways 82. These results indicated that cholangiocyte-secreted H19-exosomes played a critical role in promoting macrophage activation and hepatic inflammation in cholestatic liver diseases. The current status of little research on the communication of EVs between cholangiocytes and immune cells led us to propose that CCL-2/CCR-2 signaling may induce the release of proinflammatory EV from cholangiocytes, which further activate immune cells by a chemokine-dependent process. In addition to kupffer cells, whether EVs participate in the intercellular communication between cholangiocytes and other immune cells such as myeloid and lymphoid cells regulated by CCL2 by driving janus kinase 2 (JAK2)/STAT3, mitogen-activated protein kinases or PI3K signaling associated with cell survival remains to be further investigated.

## Immune cells-centered intercellular communication *via* EVs: a promising therapeutic option

### Delivering EVs from immune cells to hepatocytes

In addition to above hepatic parenchymal and non-parenchymal cells, the liver also recruits and activates many innate and acquired immune cells from themselves or bone marrow when injury occurs. These recruited immune cells regulate the immune/inflammatory and the tissue repair responses in the acute phase, while trigger the inflammatory and the tissue excessive repair responses alternately in the chronic inflammatory phase, evoking secondary tissue damage. Indeed, recent accumulating evidence suggests that immune cells secreted therapeutic EVs to rescue dying hepatocytes. TLR3-activated macrophages were reported to transmit the anti-fibrotic and anti-inflammatory factor miR-29 to infected hepatocytes through exosomes, thus suppressing virus replication and fibrotic liver damage 83. Recombination signal binding protein-Jκ (RBPJ) is a critical switch for Notch signaling that is responsible for the activation and differentiation of macrophages. He* et al.* demonstrated that exosomes loaded with RBPJ decoy oligodeoxynucleotides (ODNs) by tail vein administration could be primarily taken up by macrophages and blocked Notch signaling, thereby ameliorating hepatic inflammation and fibrosis in CCl_4_- or BDL-induced mouse model 84. Contradictorily, Zhang *et al.* discovered that RBPJ-overexpressed macrophages secreted abundant curative EVs to respond liver malignant diseases resulting from progressive fibrosis. When RBPJ-overexpressed macrophages were transplanted into the nude mice, the severity and volume of malignant liver lesions were prone to alleviate and shrink because exosomal Hsa_circ_0091570 from macrophages was delivered into HCC cells and acted as a ceRNA of miR-499b-5p to result in the repression of JAM3 85. Therefore, the multifaceted effect of RBPJ expressed in macrophages during liver diseases are associated with the different of clinical disease stages. We hypothesized that inhibition of RBPJ in the early stage of disease is a possible therapeutic approach to fibrotic liver diseases, while the inhibition of RBPJ in the late stage of the disease may lead to unsatisfactory treatment effect.

It is generally believed that neutrophil infiltration is associated with excessive hepatic inflammation and subsequent liver fibrosis. Contrary to what is well accepted, interestingly, Hou* et al.* identified that macrophages and neutrophil infiltration alleviated tissue fibrotic remodeling during initial phase of fibrotic response in NASH. IL-6 activated EVs biogenesis- and trafficking-related genes (*Hrs*, *Stam1* and *Vta1*) to release a large number of EVs containing miR-223 from myeloid cells *via* STAT3 activation. After *IL-6* knockout combined with high-fat diet, neutrophils and infiltrating macrophages were drastically decreased in liver, which reduced miR-223 secretion and thus upregulated TAZ expression to promote NASH and liver fibrosis 86. Soon after, He *et al.* found additional evidence about the essential role of neutrophils played in liver fibrosis. FFAs were able to activate miR-223 transcription through the up-regulation of purine-rich PU-box-binding protein 1 (PU.1) expression in neutrophils and increase the level of apolipoprotein E on neutrophil-derived EVs surface. These neutrophil-specific EVs were then selectively uptaken by steatotic hepatocytes *via* binding to low-density lipoprotein receptors (LDLR) to mitigate hepatic inflammation and fibrosis 87. Thus, EVs secreted by immune cells reduced the release of pro-inflammatory or fibrotic factors from damaged hepatocytes as well as induced apoptosis of irreparably dysfunctional hepatocytes. Moreover, whether multiple T cells, as the main acting cells of acquired immunity, have above regulatory effects on hepatocytes, is quite interesting and worth studying in the future.

### Delivering EVs from immune cells to HSCs

**Figure 6:**
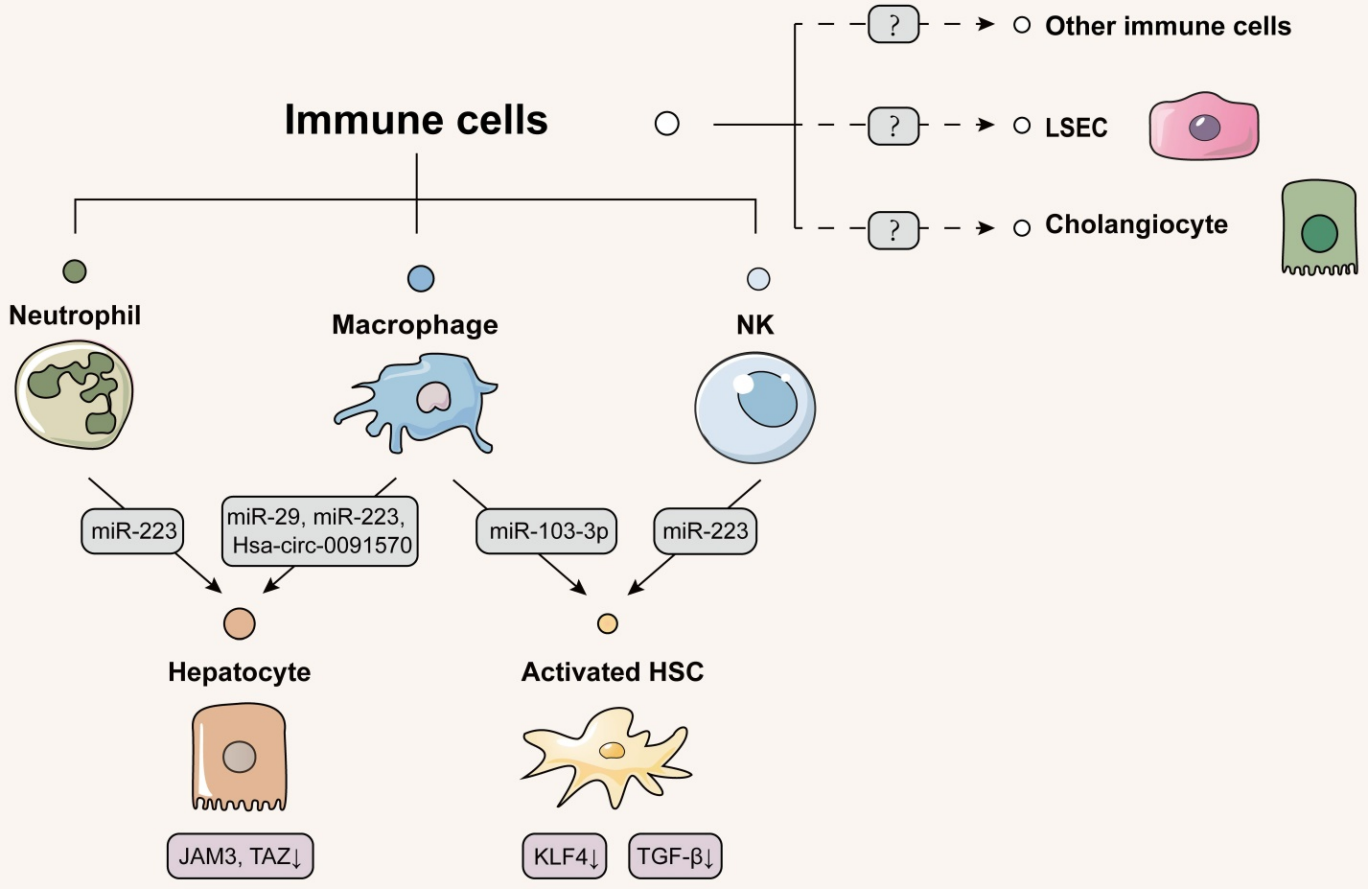
** Schematic diagram of immune cells-centered EVs network.** Immune cells primarily secrete therapeutic EVs to alleviate liver disease. Macrophages and neutrophils deliver EVs to hepatocytes to reduce the development of fibrosis and cancerous lesions. Activated HSCs receive NK cell-derived EVs to activate autophagy. Macrophages, in contrast, deliver pathogenic EVs to HSCs and promote HSC activation by inhibiting KLF4 expression. Confirmatory studies about EVs delivery from immune cells to themselves, cholangiocytes or LSECs have not been reported, which should be considered for further study. Solid lines indicate that this has now been reported and dashed lines indicate that it has not.

Emerging evidences have indicated that immune cells also release extracellular signals to directly regulate HSC activation. A recent clinical study found that NK cells were positively correlated with better liver function in HCV-associated liver fibrosis, revealing that increased NK cells might slow down the fibrogenesis 88. EVs isolated from NK cells not only distinctly mitigated TGF-β1-reduced proliferation and activation of HSCs *in vitro*, which was reversed by a specific exosome inhibitor GW4869, but also alleviated in the CCl_4_-induced fibrotic mouse model 89. Wang *et al.* further provided more direct evidence for this specific phenomenon and found that NK cells could rapidly transfer exosomal miR-233 to HSCs that suppressed autophagy by inhibiting the mRNA expression of autophagy marker ATG7, and consequently decreased the number of TGF-β-activated HSCs 90. Unlike protective effects induced by NK cells, recent research pointed out that macrophages secreted pathogenic EVs to activate HSCs and thus brought about a damaging effect on the liver. Stimulated with lipopolysaccharide, the miRNA profile of THP-1 macrophages-secreted exosomes were significantly altered with increased levels of miR-155-3p and miR-103-3p and reduced levels of miR-20a-3p and miR-106b-5p. Notably, miR-103-3p-enriched exosomes promoted the proliferation and activation of HSCs *via* the inhibition of KLF4 expression 91. Since hepatocyte-derived exosomes have been previously mentioned to inhibit KLF4 expression in HSCs, we speculate that KLF4 may be an important potential target for intercellular communication by EVs in the liver. Therefore, we conjecture whether EVs secreted by immune cells enter LSECs and activate endothelial inflammation in the liver sinusoids by regulating KLF, which can be a point for future research (**Figure 6**).

## Discussion

In this review, we comprehensively summarized both pathogenic and protective effects of EVs on different fibrotic liver diseases by describing in detail the substances encapsulated in EVs secreted or excreted by different cells in the liver, and how these substances affect the target cells in the liver (**Figure 2-6**). Based on the summary of EV transmission by specific liver cells, the 'good' or 'bad' EVs that transmit intercellular signals between two deterministic liver cells could be more obviously identified, thus helping researchers to delve into the fundamental rules of EV-mediated signaling in the liver.

Taken as a whole, there are more studies related to hepatocytes affecting HSCs and immune cells *via* EVs but only few studies about cholangiocytes and LSECs affecting other cells in the liver. On the one hand, growing experimental evidence showed that cholangiocytes injury is essential for myofibroblast differentiation of portal fibroblasts and HSCs, suggesting a non-negligible role of cholangiocytes in the pathogenesis of hepatic fibrosis 92. In addition, cholangiocytes also play an important role in the immune response of recruited immune cells by regulating the release of inflammatory cytokines and chemokines. Meanwhile, damaged cholangiocytes are also reported to express pattern-recognizing molecules to sense inflammatory mediators secreted by themselves and further regulate their survival and function 6. On the other hand, LSECs represent the first barrier orchestrating liver responses to pathogens, tissue injury and various trauma. Once injured, abnormal phenotypic changes of LSEC characterized by the loss of fenestrae and formation of large gaps and basement membrane contribute to liver fibrosis progression. Of note, the above two cell types are physically close to each other and have been reported to release pathogenic substances into the hepatic environment, however, few studies have made direct evidence about which targeted liver cells are these substances released into. Moreover, it also remains to be determined whether these damaged cells interfered with the process of cellular communication by altering their own physiological properties or promoting the release of pathological signals. Therefore, we suggest that more researchers should pay greater attention on the intercellular communication among cholangiocytes, immune cells and LSECs in the liver. At the same time, we speculate that the reason why these cells have been troublesomely explored may be related to the less content and distribution in the liver and the technical difficulty of the extraction method. Therefore, optimizing current available extraction techniques or making full use of single-cell sequencing may provide effective means of assisting EV-related research in the future.

Among numerous studies reviewed here, hepatocytes represent the 'spotlight cells' of cell-to-cell communication in the liver. Once liver injured, hepatocytes first respond to harmful stimulus and appear structural and functional disorders. Hepatocyte injury is typically driven by cell apoptosis or necrosis that is associated with increased mitochondrial membrane permeability and the activation of death receptors. Ruptured hepatocyte may initiate the release of multiple damage signals, and a portion of which can be delivered to normal hepatocytes and non-parenchymal cells *via* EVs. Since there are far more hepatocytes than other liver cells, an imbalanced ratio of injury signals is flooded to the receptor cells, which allows various liver cells to rapidly receive stimulus and then activates fibrotic signaling cascades. The elimination of EVs from damaged hepatocytes or the enhancement of EVs production artificially in normal hepatocytes might provide effective methods for treating fibrotic liver diseases. Although the effects of immune cells have been defined at two separate states, including anti-inflammatory and pro-inflammatory states, the interaction between immune cells and hepatocytes mainly results in the alleviation of fibrotic liver injury. In summary, selective amplification of intercellular signals released by immune cells *via* EVs is a new alternative way to reduce fibrotic liver diseases.

MiRNAs regulate the expression of target genes after transcription and participate in the occurrence of many diseases by binding to the 3'untranslated region or open reading frame transcriptional direction of their target mRNAs. Under the normal condition, the susceptible degradation and inefficient cellular uptaken characteristics of miRNA make it difficult to reach the receipt cells. However, because of neutral sphingomyelinase 2 or (and) heterogeneous nuclear ribonucleoproteins activation by its specific sequences 93, 94, miRNAs can be entrapped within EVs with higher stability, and can be effectively delivered to distant receptor cells and regulate multiple biological processes. In addition, the ability of intracellular miRNA to be loaded into exosomes was also enhanced owing to the direct binding of synaptotagmin-binding cytoplasmic RNA-interacting protein (SYNCRIP) to specific miRNAs enriched in exosomes sharing hEXO motif 95. However, the mechanisms by which miRNAs are sorted into EVs or retained in cells remain largely unknown. Recently, researchers have made a breakthrough in this field by demonstrating that miRNAs contain sequencing sequences that determine their secretion or cell retention in small EVs (sEVs), and that different cell types use specific sequencing sequences to determine the corresponding sEV miRNA profiles. Insertion or deletion of these sequencing sequences in miRNAs can increase or decrease the intracellular production or secretion of exosomes and other sEVs, therefore, the code possessed by miRNAs is an important factor in linking circulating exosomal miRNAs to tissues of origin 96. Similar to other diseases, the contents of exosome transmitted between cells are mainly miRNAs and few easily decomposed proteases in the pathological environment of fibrotic liver diseases. Contrasting the available studies, we surprisingly find that miRNAs are primarily focusing on miR-103, miR-1297, miR-192, miR-25, miR210, miR-233, miR-19, miR-30 and miR-335, especially for miR-233 and miR-192, since the former is a pivotal miRNA of lipid metabolism and hepatic inflammation and the latter is expressed at higher level in the liver. Therefore, researchers can shed light more on miRNA to explore contents of EVs transmitting biological signals between liver cells in future studies.

Prior studies have shown that plasma EVs from healthy donors protect myocardium from ischemia-reperfusion injury 97. In addition, serum EVs have been reported to maintain homeostasis under normal conditions and also alleviate acute hindlimb ischemic injury in mouse model 98, suggesting the rich therapeutic value of serum EVs for treating multiple pathological injuries. In recent years, since EVs function as conduits for intercellular molecular transfer, the therapeutic effects of EVs have increasingly aroused the interests of researchers in liver diseases. It has been reported that EVs from healthy donors possess anti-fibrotic properties. EVs derived from normal mouse serum have been reported to inhibit CCl_4_ or thioacetic acid-induced fibrotic liver injury by inhibiting fibrogenic- and HSC activation-related gene expression in mice 97. Similarly, human serum EVs have been reported to have similar therapeutic effects as mouse serum EVs 97. Notably, the expression levels of several miRNAs were found to be relatively high in EVs based on summary of reviewed above. We thus speculate that the suppression of miRNAs in EVs results in a therapeutic action targeting HSC fibrogenesis. So, it will be of great value to understand the function of EVs in hepatic intercellular communication and their potential as carriers of biomarkers to aid disease diagnosis and prognosis. At the same time, some researchers have found that EVs derived from red blood cells are natural liver drug carriers because of their characteristics on liver accumulation 99. Exosomal membrane protein CD9 was fused with RNA binding protein to encapsulate target miRNAs in exosomes and relief liver injury caused by CCl_4_* in vivo* 100, suggesting that the construction of artificial liver targeted EVs may be applied in clinical practice. With the rapid development of isolation, detection and artificially-modification technology of EVs, the researches and clinical applications of EVs will be much more in-depth, revealing the irreplaceability of EV in the treatment of fibrotic liver disorders and their complications.

## Conclusion

In the current review, we provide an update information on the contents of the intercellularly delivered EVs as well as their roles between different liver cells. Furthermore, our work points out the shortcomings of current EV research and proposes approaches for improving the rigorousness of future studies. We expect that these intercellular EVs will have the potential to become diagnostic and therapeutic targets in the near future, which will greatly influence the scenery of hepatology and other medical specialties.

## Abbreviations

BMDM: bone marrow-derived macrophages
CXCL10: C-X-C motif ligand 10
DAMPs: damage-associated molecular patterns
ECM: extracellular matrix
EVs: extracellular vesicles
HSC: hepatic stellate cell
HUVECs: human umbilical vein endothelial cells
IFN: interferon
IL-17A: interleukin-17A
LSEC: liver sinusoidal endothelial cell
mtDNA: mitochondrial DNA
MMP2: matrix metalloproteinase 2
MVBs: multivesicular bodies
PDGF: platelet-derived growth factor
PTEN: phosphate and tension homology deleted on chromosome ten
SMAD: small mothers against decapentaplegic
STAT3: signal transducer and activator of transcription 3
TLR3: toll-like receptor 3
VEGF: vascular endothelial growth factor
